# Tryptase Promotes Atherosclerotic Plaque Haemorrhage in ApoE-/- Mice

**DOI:** 10.1371/journal.pone.0060960

**Published:** 2013-04-03

**Authors:** Xiuling Zhi, Chen Xu, Hao Zhang, Dai Tian, Xiaobo Li, Yanxia Ning, Lianhua Yin

**Affiliations:** 1 Laboratory of Medical Molecular Biology, Teaching Center of Experimental Medicine, Shanghai Medical College, Fudan University, Shanghai, China; 2 Department of Pathology, Zhongshan Hospital, Fudan University, Shanghai, China; 3 Department of Physiology and Pathophysiology, Shanghai Medical College, Fudan University, Shanghai, China; Virginia Commonwealth University, United States of America

## Abstract

Tryptase, the most abundant mast cell (MC) granule protein, plays an important role in atherosclerosis plaque development. To test the hypothesis that tryptase participates directly in atherosclerosis plaque haemorrhage, the gene sequence and siRNA for tryptase were cloned into a lentivirus carrier and atherosclerosis plaque haemorrhage models in ApoE-/- mice were constructed. After a cuffing-cervical artery operation, the mice were randomly divided into 6 groups. Hematoxylin and eosin(HE) staining showed that the cervical artery plaque area was much larger in the tryptase overexpression group compared to the other groups, and there was greater artery stenosis. The artery stenosis from the cuff-side in all groups was more than 90%, except the siRNA group. Tryptase promotes plaque haemorrhage distinctively because 50% of the mice in the tryptase overexpression group had plaque haemorrhage, while only 10% in the siRNA group did. The immunohistochemistry of the cervical artery plaque showed that plasminogen activator inhibitor-1 (PAI-1) expression was the lowest while tissue plasminogen activator (tPA), CD31, CD34 and VEGF was the highest in the tryptase overexpression groups. This observation was completely contrary to what was observed in the siRNA group. Tryptase promoted bEnd.3 cell growth, migration and capillary-like tube formation, which suggests that tryptase can promote microvessel angiogenesis. PAI-1 expression was inhibited, while tPA expression was increased by tryptase in bEnd.3 cells. Our in vivo and in vitro studies suggest that trypase can promote atherosclerotic plaque haemorrhage by promoting angiogenesis and regulating the balance of PAI-1 and tPA. Thus, regulating tryptase expression in MCs may provide a potential target for atherosclerosis treatment.

## Introduction

The incidence of acute cardiovascular events caused by atherosclerosis has greatly increased over what it was before. Understanding the process of plaque haemorrhage is crucial given the close relationship with plaque angiogenesis [1]. Therefore, understanding the mechanisms by which plaque angiogenesis and haemorrhage occur may ultimately help prevent the transition from a stable to an unstable lesion.

Mast cells have been demonstrated to be critically involved in the pathogenesis of atherosclerosis and acute cardiovascular syndromes [2]. Activated mast cells degranulate and release many types of mediators including cytokines, chemokines, growth factors, vasoactive substances and proteolytic enzymes. These mediators can profoundly influence the inflammatory response and angiogenesis within atherosclerotic plaques [3], [4]. Tryptase, the dominant secretary granular protein in human mast cells, is a tetrameric neutral serine protease with multiple biological functions and plays important roles in cardiovascular diseases, including atherosclerosis [5].

Kinoshita et al. [6] have reported that human tryptase may promote vascular inflammation by increasing inflammatory mediator production of the monocyte chemoattractant protein-1 (MCP-1) and interleukin-8 (IL-8) in human endothelial cells. Our preliminary study also demonstrated that tryptase induces the PI3K-PKB pathway through protease-activated receptor-2 (PAR-2) and up-regulates IL-8 expression in ECV304 cells [7]. Tryptase could also activate peripheral blood mononuclear cells causing the synthesis and release of tumour necrosis factor-alpha (TNFα), IL-6 and IL-1 beta [8]. Proinflammatory factors, such as IL-8, are also angiogenic factors. In addition to its proinflammatory effects, in vitro studies recently identified tryptase as a major factor in mast cell-mediated lipid uptake because it enhanced foam cell formation in THP-1 macrophages by suppressing LXRα activation in a PAR-2 dependent manner [5]. Tryptase may reduce the efflux of cholesterol from macrophages by depleting high density lipoprotein (HDL) and thus increase the formation of foam cells [9].

Moreover, tryptase could function as a potent angiogenic factor. Blair et al. (1997) have reported that tryptase is a mitogen for human dermal microvascular endothelial cells and causes a significant augmentation of capillary growth, which is suppressed by specific tryptase inhibitors [10]. Recently, tryptase has been reported to correlate with tumour angiogenesis, such as in early breast cancer [11], lung cancer [12] and B-cell chronic lymphocytic leukaemia [13]. However, there are few reports that demonstrate the relationship between tryptase and intraplaque angiogenesis.

Pathologic examination of unstable lesions has demonstrated that intraplaque haemorrhage is associated with an increased density of microvessels and inflammation [1]. It has also shown that tryptase is associated with both angiogenesis and inflammation, which suggests tryptase may play an important role in atherosclerotic plaque stability. Although tryptase release was observed around the calcified deposits and was a common feature in advanced lesions with fissure, haemorrhage and thrombus formation [14], the role of tryptase in atherosclerotic plaque haemorrhage is still unclear. In this study, we used lentivirus carriers to study the effect of tryptase on plaque haemorrhage in vivo and in vitro. The expression of plasminogen activator inhibitor type 1 (PAI-1) and tissue plasminogen activator (tPA) was also analysed.

## Materials and Methods

### Animals and cells

Male apoE-/- mice (Jackson Labs,USA) were ten weeks old at the time of entry into the study. The animals received a western type diet containing 0.25% cholesterol and 15% lard to the end of the experiment [15]. Approval for the study was received from Experimental Animal Ethics Committee, Fudan University Shanghai Medical College (Permit Number: 20110307-027).

The bEnd.3 mouse brain endothelial cell line (ATCC-No. CRL-2299) and mouse mastocytoma cell line P815 (ATCC-No. TIB-64) were cultured according to the supplier's instructions in DMEM supplemented with 10% FBS. Cells were maintained at 37°C and 5% CO_2_ in a humid chamber.

### Construction of lentivirus carriers for tryptase expression and tryptase shRNA expression

The tryptase expression vector Lent-GFP-tryptase lentivirus, control lentivirus Lent-GFP, tryptase siRNA expression vector Lent-RFP-siRNA lentivirus and control lentivirus Lent-RFP-controlsiRNA were constructed by Shanghai Minghong Biology CO., LTD.

Briefly, full sequence of mouse tryptase(mouse mast cell protease,mMCP6, NM_010781) was cloned and connected to the lentiviral vector pLV-UbC-GFP-3FLAG with a hUbC promoter. In the other hand, three siRNA expression plasmids (named as siRNA1, siRNA2 and siRNA3 respectively) were designed and constructed according to the tryptase gene sequence. The control siRNA sequence was a randomly scrambled sequence that is not found in the mouse, human, or rat genome databases. Lent-RFP-siRNA lentivirus and control lentivirus Lent-RFP-controlsiRNA were constructed after the effect of the 3 siRNA plasmids was determined.

### P815 cell transfection

First, 3×10^5^ P815 cells/well were plated in 6-well plates and cultured with DMEM (Dulbecco's modified Eagle's medium) (Gibco, USA) containing 10% (v/v) FBS (foetal bovine serum) (Gibco, USA) at 37°C for 24 h before transfection. The cells were then divided into 3 groups with 2 wells per group: Lent-GFP-tryptase lentivirus-treated group, Lent-GFP control lentivirus-treated group and blank control group (equal volume of PBS). The cells were observed by a fluorescent microscope 24 h after transfection. The percentage of positive cells in 10 fields was counted to evaluate the transfection efficiency. To analyse the effect of Lent-GFP-tryptase lentivirus on tryptase gene expression, the tryptase mRNA level in P815 cells 24 h after they were treated with lentivirus was measured.

To determine the effect of the 3 siRNA plasmids, siRNA plasmid and Lipofectamine 2000 transfection mixtures were added to each well of P815 cells 24 h after Lent-GFP-tryptase lentivirus treatment. We measured the tryptase mRNA level in P815 cells 24 h after treatment with siRNA plasmids. One of the 3 siRNA plasmids which decreased tryptase mRNA expression most effectively was used to construct the Lent-RFP-siRNA lentivirus.

### Preparation of conditioned medium and analysis of tryptase activity

After lentivirus treatment, 3 groups of P815 cells (Lent-GFP-tryptase lentivirus treated, Lent-GFP control lentivirus treated and the blank control) were maintained in DMEM medium containing 10% (v/v) FBS for 24 h. The cells were then washed with PBS 3 times and cultured in serum-free DMEM medium for an additional 24 h. Fifteen min after treatment with mast cell-degranulating agent compound 48–80 (1 µg/mL, Sigma, USA), the culture media was collected, centrifuged, filtered and named as positive, mock and blank P815-conditioned media, respectively. The same volume of serum-free DMEM medium without cells was also cultured for 24 h and treated with compound 48–80 as “correct control”. Tryptase activity in the P815 cell medium was assayed using an enzymatic procedure.

Tryptase was assayed by hydrolysis of the synthetic substrate N-p-tosyl-Gly Pro-Lys-p-nitroanilide (Item T-6140, Sigma, USA). Release of p-nitroaniline was followed at 410 nm over 15 min at 37°C with a “Cobas Bio” centrifugal analyser. The reaction mixture contained 100 µL 10 mM Tris buffer pH 7.4 supplemented with 5 mM CaCl2 and 100 mg/L bovine lung heparin (Item H-4898, Sigma, USA). The assay was initiated by addition of the peptide substrate to give a final peptide concentration of 0.6 mM.

### Cell viability assay

Cell viability was determined by an MTT assay as originally described by Mosmann [16]. In brief, bEnd.3 cells were plated at a density of 10^3^cells/well onto 96-well tissue culture plates and incubated with positive, mock and blank p815-conditioned culture medium at 37°C for 0, 24, 48, and 72 h. Then, 10 µl of MTT reagent (5 mg/ml) was added to each well, and the plates were incubated for another 4 h. The medium was removed, and the wells were rinsed twice with PBS. To each well, 100 µl of dimethyl sulphoxide (DMSO) was added at room temperature to dissolve the formazan crystals. The absorbance was measured at 490 nm with a Spectramax3000 plate reader (Molecular devices, Sunnyvale, CA).

### Migration and tubular formation assay

Cell migration was assayed in a 24-well transwell migration chambers (8-µm pore size; Corning, Lindfield, NSW, Australia). First, bEnd.3 cells (5×10^4^/200 µl) were seeded into duplicate chambers and allowed to migrate for 18 h at 37°C in conditioned culture medium. In the lower compartments of the chambers, 500 µl of DMEM medium containing 10% FBS was added. Cells remaining on the upper side of the insert were removed by wiping with a cotton swab. The cells that migrated through the filters were fixed, stained with Giemsa, photographed, and counted. Migration assays were completed at least three times in duplicate wells.

In the tubular formation assay, bEnd.3 cells were harvested by trypsinisation, resuspended in conditioned culture medium and plated (30,000 cells/well; 500 µL of the suspension) into 24-well plates that were coated with 100 µL of 1 mg/mL Matrigel. Tubule formation was quantified 24 h after plating by viewing the cells at ×200 magnifications in a blinded manner.

### Real-time PCR

Total RNA was extracted from cells with TRIzol reagent (Invitrogen, USA). Then, 2 µg was used to synthesise cDNA with the Superscript First-Strand Synthesis Kit (Promega, USA) following the manufacturer's protocols. The real-time PCR reaction kit contained 0.2 µM sense primer, 0.2 µM antisense primer, 12.5 µl SYBR Green I (Toyobo, Osaka, Japan), and 5 µl of previously synthesised cDNA in a total volume of 25 µl. A pair of GAPDH primers in identical reactions were used to control the starting template. The sense and antisense primers were (5′- ACAGCCGCATCTTCTTGTGCAGTG- 3′) and (5′-GGCCTTGACTGTGCCGTTGAATTT -3′), respectively. The target genes detected were mouse tryptase (forward: 5′-AGTAAGTGGCCCTGGCAGGTGAGCC-3′, reverse: 5′-GGTCCCCATAGTATAGATACTGCTC-3′), mouse tPA (forward: CTGAGGTCACAGTCCAAGCA, reverse: ACAGATGCTGTGAGGTGCAG) and mouse PAI-1 (forward: GTCTTTCCGACCAAGAGCAG, reverse: ATCACTTGGCCCATGAAGAG) [17].

### Western blot analysis

Western blot analysis was performed as previously described [18]. Briefly, cells were lysed and the supernatants were collected. Then, 30 µg of protein was loaded to a 10% SDS-PAGE to detect PAI-1 and tPA expression levels with GAPDH as a protein loading control. Primary antibodies against PAI-1 and tPA were purchased from Santa Cruz Biotechnology.

### Right common carotid collar placement and experimental design

Mice were anaesthetised by intraperitoneal injection of sodium pentobarbital (50 mg/kg) and subjected to carotid collar placement. Collars (length: 3 mm, internal diameter: 0.38 mm, external diameter: 2.2 mm) were made from Tygon® tubing and were positioned around the right carotid artery. Then, the collar's axial edge was approximated by placement of 3 circumferential silk ties [19]. The contralateral left carotid artery was sham-operated to serve as the intra-animal control. Subsequently, the entry wound was closed, and the animal was returned to its cage for recovery from the anaesthesia.

Following carotid injury, the animals were maintained on a western diet and randomly divided into the following 6 groups (n = 10): Lent-GFP-tryptase, Lent-GFP, Lent-RFP-siRNA, Lent-RFP-controlsiRNA, blank degranulation and blank control. Four weeks after surgery, 4 groups of mice were treated with Lent-GFP-tryptase lentivirus, control lentivirus Lent-GFP, Lent-RFP-siRNA lentivirus or control lentivirus Lent-RFP-controlsiRNA at 1×10^8^ TU/mice by tail intravenous injection. Six weeks after surgery, 3 groups of mice with Lent-GFP-tryptase, Lent-GFP or blank degranulation were intraperitoneally injected with the mast cell degranulator compound 48-80 (Sigma, USA) 0.5 mg/kg, and the other 3 groups with Lent-RFP-siRNA, Lent-RFP-controlsiRNA and the blank control were intraperitoneally injected with an equal volume of the mast cell inhibitor, cromolyn sodium (Sigma,USA) 10 mg/kg every other day for 10 times. Ten weeks after surgery, all mice were sacrificed to harvest tissue specimens.

### Tissue harvesting and preparation

The mice were anaesthetised, and blood was collected from the retro-orbital plexus. Serum was collected and stored at −70°C until the determination of the serum parameters as described below. Subsequently, both common carotid arteries were removed and fixed in neutral buffered formalin. Then, frozen tissue sections were prepared for immunohistochemical analysis.

In the preliminary test, 2 apoE-/- mice were underwent right common carotid collar placement surgery and were maintained on a western type diet. Four weeks after surgery, one mouse was treated with Lent-RFP-controlsiRNA at 1×10^8^ TU/mice by tail intravenous injection, and the other as blank control. Two weeks after injection, the carotid arteries were removed and embedded within the optimum cutting temperature compound (O.C.T compound, Sakura Finetek, Inc., Torrance, CA) without being fixed in neutral buffered formalin. Frozen tissue sections were then prepared and immediately examined with a fluorescence microscope.

### Determination of blood parameters

The levels of triglycerides (TG), total cholesterol (TC), HDL cholesterol (HDL-C) and low-density lipoprotein cholesterol (LDL-C) in serum were measured by enzymatic methods according to the manufacturer's instructions using the detection kit purchased from Rongsheng Biotechnology Company Ltd. (Shanghai, China).

### Histology, special stain and immunohistochemistry

Ten-micrometre tissue sections were routinely stained with haematoxylin and eosin (HE).All sections were stained with Sudan III, Prussian blue and Toluidine Blue according to the standard protocols established by our laboratory. Corresponding sections on separate slides were stained with rabbit polyclonal antibodies against tryptase (FL-275, Santa Cruz,USA), PAI-1 (SERPINE1 Antibody, PTG,USA), tPA (PLAT Antibody, PTG,USA), the endothelial cell specific antigen CD31 (M-185, Santa Cruz,USA), CD34(H-140, Santa Cruz,USA) and VEGF(A-20, Santa Cruz,USA). The slides were incubated with primary antibody for l h at room temperature. Then, goat anti-rabbit IgG peroxidase conjugate (BD, USA) was used as secondary antibodies (1 h incubation at room temperature) with 3,3-diaminobenzidine(DAB) as enzyme substrates. Double stain of tryptase and GFP (F56–6A1, mouse polyclonal to GFP, Santa Cruz, USA) was also performed using 3-amino-9-ethylcarbazole (AEC) and DAB as substrates respectively.

Quantification of immunohistochemistry staining was analyzed by using Image-Pro Plus 5 (Media Cybernetics, Bethesda, MD) including 3 parameters: density mean, area sum, and integrated optical density (IOD). Mean density = (IOD SUM)/area.

### Statistical analysis

The results were expressed as the mean±standard deviation (SD) of the individual specimen measurements. Values of *P*<0.05 were considered to be statistically significant. ANOVA and the Student-Neuman-Keuls post test were performed to compare the two groups.The statistical analysis was performed using SPSS version 11.0.

## Results

### Effects of tryptase lentivirus carrier and tryptase siRNA lentivirus carrier

**Figure 1:**
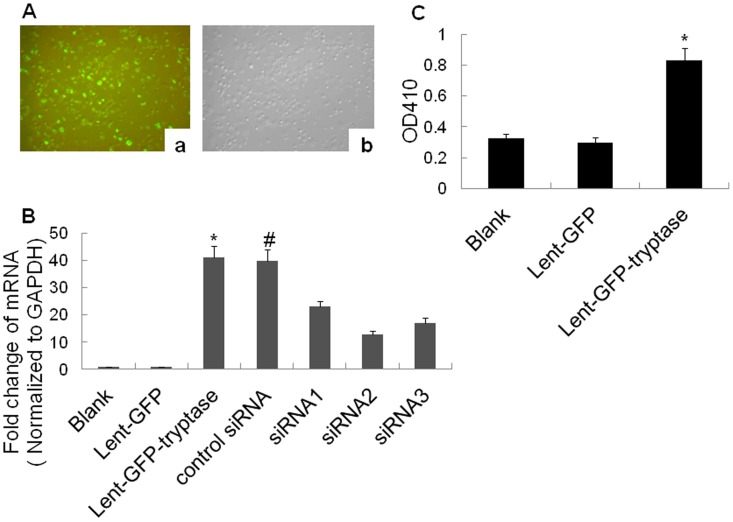
Constructions of tryptase lentivirus carrier and tryptase siRNA lentivirus carrier. A. Transfection efficiency detected by fluorescent microscopy. a. GFP-emitted green fluorescence. b. Same fields of vision under an optical microscope(×100). More than 80% cells were infected with lent-GFP at a dose of MOI 10 after 48 h treatment. B. Tryptase mRNA level by real-time PCR. The Lent-GFP-tryptase lentivirus significantly increased the tryptase mRNA level in the P815 cells. *P<0.01 vs. blank and Lent-GFP (n = 3). Additionally, siRNA2 plasmid decreased P815 cell tryptase expression at the mRNA level most significantly. *P<0.01 vs. siRNA2, #*P*>0.05 vs. Lent-GFP-tryptase (n = 3). C. Tryptase activity assay. The Lent-GFP-tryptase lentivirus increased tryptase activity in the P815 cell medium significantly. *P<0.01 vs. blank and Lent-GFP (n = 3).

To assess the efficiency of Lentiviral transduction, P815 cells were plated at a density of 1×10^5^ cells/well into 24-well plates and infected with lent-GFP at various multiplicities of infection (MOIs) 24 h after seeding. After 48 h, GFP-expressing cells were detected by fluorescence microscopy (Olympus, Japan). We found that P815 cells exhibited high Lentiviral transduction efficiency, because more than 80% cells were infected with lent-GFP at a dose of MOI 10. (Fig. 1A).

We analysed the expression of tryptase at the mRNA level in P815 cells using real-time RT-PCR. Our results demonstrated that Lent-GFP-tryptase lentivirus significantly increased tryptase mRNA expression in P815 cells, while siRNA plasmids reversed the effect of Lent-GFP-tryptase lentivirus. The plasmid “siRNA2” targeting tryptase sequence: GCTCCTCTCTTTGAACAGGAT decreased tryptase mRNA expression most effectively and was used to construct Lent-RFP-siRNA lentivirus. (Fig. 1B)

For the tryptase activity assay, the culture medium from the P815 cells was collected and centrifuged, 15 min after treatment with compound 48–80. T-6140 was used to measure tryptase activity. The results showed that Lent-GFP-tryptase lentivirus significantly increased tryptase activity in P815 cell culture medium (Fig. 1C).

### The expression efficiency of lentivirus in apoE-/- mice by tail vein injection

**Figure 2:**
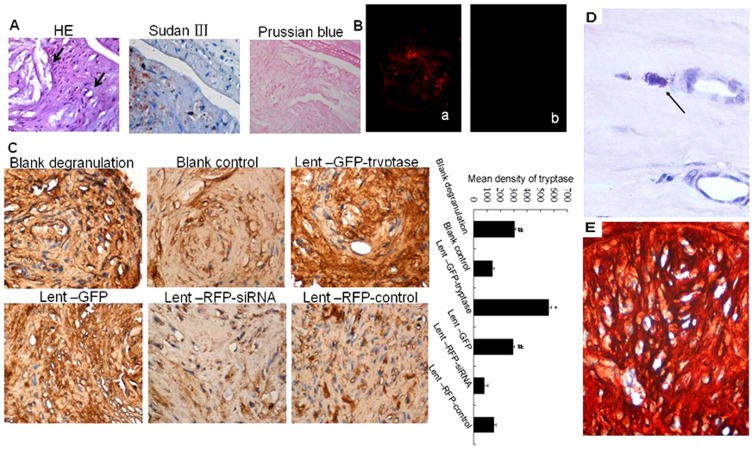
Effects of lentivirus carriers in apoE-/- mice by tail vein injection. A. Formation of atherosclerotic plaques in cervical arteries. Black arrows in the HE section (×400) show the cholesterol crystals (the upper arrow) and foam cells(the lower arrow). Lipids in the plaque show orange-red by Sudan III stain(×400). Prussian blue stain shows negative results in the plaque (×400). B. ApoE-/- mouse cervical artery frozen section observed under a fluorescent microscope. Red fluorescence can be observed in the plaque after letivirus injection. a. RFP expression in the plaque of the Lent-RFP-controlsiRNA mouse detected under a fluorescent microscope (×100). b. No RFP expression was observed in the plaque of the blank control mouse (×100). C. Immunohistochemical stain of tryptase stain in the atherosclerotic plaques. Tryptase expression showed the highest level in the Lent-GFP-tryptase group, while the lowest level was found in the Lent-RFP-siRNA group. *P<0.01 vs. other groups (n = 5). #*P*<0.05 vs. blank control, Lent-RFP-siRNA and Lent-RFP-control (n = 5).D. Mast cell aggregation in the atherosclerotic plaques. Toluidine Blue staining showed activated and degranulating mast cells (arrow, ×800). E. Double stain of tryptase and GFP in the plaques (×400). The stain of tryptase and GFP using AEC or DAB as enzyme substrates showed red or brown respectively. The colour of red brown showed in the plaque because of the two colours overlapped, which indicated the co-localization of the two proteins.

Atherosclerotic plaques formed in carotid arteries of both sides in apoE-/- mice. Cholesterol crystals and foam cells were observed in HE stained artery sections, and the lipid deposits wthin the plaques were confirmed by the special stain Sudan III (Fig. 2A). Negetive results of Prussian blue stain also confirmed the plaque formation by ruling out the possibility of organized thrombi (Fig. 2A).

There was RFP expression in the atherosclerotic plaques from apoE-/- mice 2 weeks after Lent-RFP virus injection at 10^8^ TU/mice into the tail vein (Fig. 2B). Toluidine Blue stain showed mast cells aggregation in the plaques (Fig. 2D). The double stain showed that tryptase and GFP co-localized in the plaques (Fig. 2E). In atherosclerotic plaques of the 6 groups, tryptase expression was the highest in the Lent-GFP-tryptase group, while the lowest in the Lent-RFP-siRNA group (Fig. 2C). The results suggest that the constructions of lentivirus could increase or decrease tryptase expression in atherosclerotic plaques.

### Serum lipid levels of apoE-/- mice

After eight weeks of a western diet, blood TC, TG, HDL-C and LDL-C concentrations in the six groups was tested. The results showed there were no significant differences in the lipid levels between the groups (Table 11).

**Table 1:** The concentration of serum TC, TG, HDL-C and LDL-C in ApoE-/-mice (mmol/L).

Group	TC	TG	HDL-C	LDL-C
Blank degranulation	30.26±6.31	1.80±0.35	0.47±0.11	5.99±0.73
Blank control	33.98±7.36	1.74±0.41	0.50±0.15	7.26±0.91
Lent-GFP-tryptase	36.30±6.81	1.99±0.37	0.46±0.07	7.84±0.94
Lent-GFP	31.16±4.92	1.96±0.53	0.44±0.05	8.1±0.99
Lent-RFP-siRNA	33.14±5.73	1.76±0.50	0.41±0.03	7.04±0.75
Lent-RFP-control	30.92±6.52	1.65±0.19	0.50±0.04	6.91±0.64

There's no difference between all groups. n = 10.

### Overexpression of tryptase promoted plaque haemorrhage

**Figure 3:**
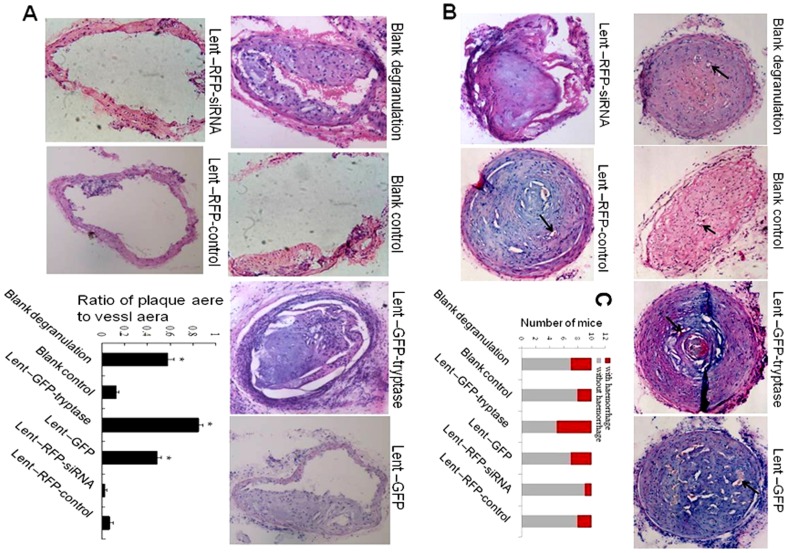
HE stains. A. HE stain on the uncuffing side of the cervical artery (×100). Compound 48–80 and tryptase increased plaque area and artery stenosis. *P<0.01 vs. blank control, Lent-RFP-siRNA and Lent-RFP-control (n = 5). B. HE stain of the cuffing side of the cervical artery (×100). Tryptase promoted plaque haemorrhage and angiogenesis distinctively. Plaque haemorrhage: red blood cell extravasation directed by black arrows. The cuffing-side artery stenosis was serious, being greater than 90% in all groups, except the lent-RFP-siRNA group. C. Number of mouse with plaque haemorrhage. 50% of mice in Lent-GFP- tryptase group have plaque hemorrhage, while only 1 mouse in siRNA group. *P<0.01vs other groups. (n = 10).

HE staining of corresponding common carotid arteries of the sham-operated mice (uncuffing-side) showed that overexpression of tryptase significantly increased carotid plaque area and the degree of carotid artery stenosis, while carotid plaque area was the smallest in the tryptase interference group compared to other experimental groups. Plaque area and artery stenosis were more prominent in the blank degranulation group than the blank group treated with mast cell inhibitor, indicating that the mast cell degranulation agent compound 48–80 has a similar role to tryptase, but the effect is weaker (Fig. 3A).

HE staining of the cuffing-side artery plaque showed that the overexpression of tryptase significantly promoted plaque angiogenesis and plaque haemorrhage (indicated by a black arrow). Artery stenosis of the cuffing-side groups was severe, being greater than 90% in all groups, except the lent-RFP-siRNA group. Additionally, 50% of the arteries with collar placement showed plaque haemorrhage in the Lent-GFP-tryptase group, while only 10% in the siRNA group showed plaque haemorrhage (Fig. 3B, C).

### Tryptase reduces PAI expression and promotes tPA expression in atherosclerotic plaques

**Figure 4:**
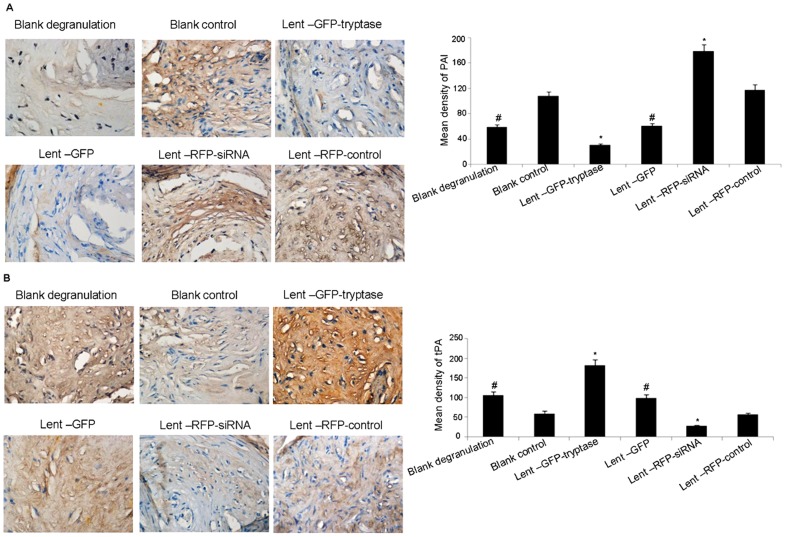
Immunohistochemical stain of PAI-1 and tPA. A. Tryptase reduced PAI expression in the atherosclerotic plaques. *P<0.01 vs. the other groups. #P<0.05 vs. control siRNA and blank control (n = 5). B. Tryptase promoted tPA expression in the atherosclerotic plaques. *P<0.01 vs. the other groups. #P<0.05 vs. control siRNA and blank control (n = 5).

The staining intensity and size of the PAI staining in the Lent-GFP-tryptase group was significantly lower than that in the other groups, while the Lent-RFP-siRNA group showed the highest staining intensity (Fig. 4A). In contrast, the staining intensity and size of tPA in the Lent-GFP-tryptase group was significantly higher than that in the other groups, while the Lent-RFP-siRNA group was the lowest (Fig. 4B). PAI expression was lower in the blank degranulation group than in the blank control group, while tPA expression showed the opposite, indicating that the mast cell degranulation agent compound 48–80 has a similar role to tryptase, with a weaker effect.

### Tryptase promotes CD31, CD34 and VEGF expression in atherosclerotic plaques

**Figure 5:**
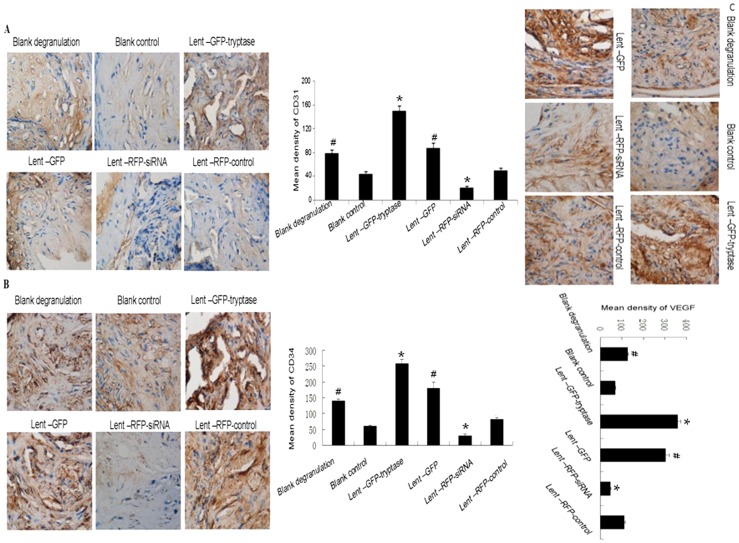
Immunohistochemical stain of CD31, CD34 and VEGF. A. Tryptase promoted CD31 expression in the atherosclerotic plaques. Mean density of CD31 staining in the Lent-GFP-tryptase group was significantly higher than the other groups, while the Lent-RFP-siRNA group was the lowest. *P<0.01 vs. the other groups. #P<0.05 vs. control siRNA and blank control (n = 5). B and C. CD34 and VEGF staining. Tryptase promoted CD34 and VEGF expression in the atherosclerotic plaques. The expression pattern in six groups was similar to CD31.*P<0.01 vs. the other groups. #P<0.05 vs. control siRNA and blank control (n = 5).

CD31-mediated endothelial cell-cell interactions play a major role in angiogenesis. Studies have shown CD31 to be a superior marker in human angiogenesis compared to factor VIII. CD34 is also an important endothelial marker and its combination with CD31 has been proved to be reliable for angiogenesis evaluation. In vivo VEGF induces angiogenesis as well as permeabilization of blood vessels, and plays a central role in the regulation of vasculogenesis. The mean density of CD31, CD34 and VEGF in the Lent-GFP-tryptase group was significantly higher compared to the other groups, while the Lent-RFP-siRNA group was the lowest (Fig. 5). The results suggest that tryptase can promote plaque angiogenesis, possibly by regulating VEGF level.

### The effect of tryptase on angiogenesis in vitro

**Figure 6:**
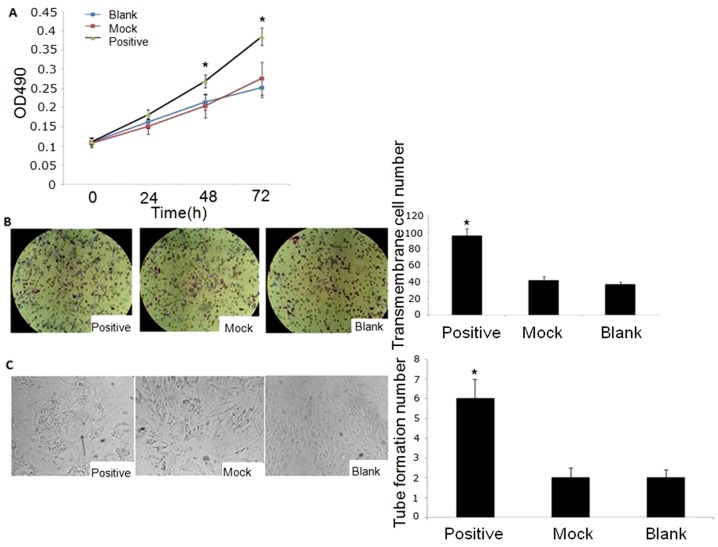
The effect of tryptase on angiogenesis in bEnd.3 cells. A. MTT assay. Tryptase promoted bEnd.3 cell growth. *P<0.01 vs. mock and blank groups (n = 3). B. Migration assay using Transwell chambers. The bEnd.3 cells were cultured with P815-conditioned medium. Tryptase promoted bEnd.3 cell migration. *P<0.01 vs. the mock and blank groups (n = 3). C. Capillary-like tube formation assay. *P<0.01 vs. the mock and blank groups (n = 3).

Using the murine microvascular endothelial cell line bEnd.3 and 3 different types of P815-conditioned media, the effect of tryptase on angiogenesis in vitro was analysed. MTT assay allows assessing the viability and the proliferation of cells. As Fig6A shown, the cellular viability was significantly increased at 48 and 72 h in positive medium (Lent-GFP-tryptase lentivirus treated P815-conditioned medium) which has a higher tryptase activity. Fig6B and C showed positive conditioned medium increased the migration and tubular formation ability of bEnd.3 cells. Our data suggest that tryptase promotes angiogenesis in vitro (Fig. 6).

### Tryptase effect on PAI and tPA expression in bEnd.3 cells

**Figure 7:**
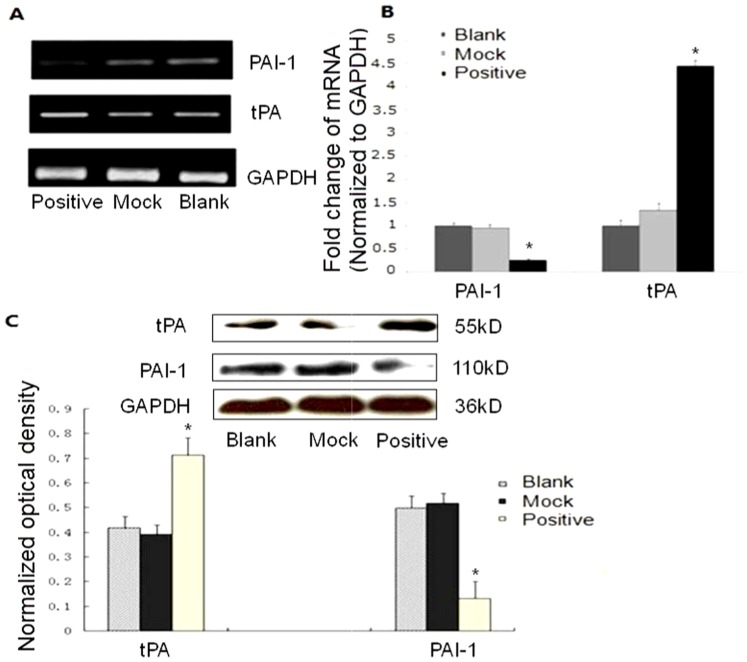
The effect of tryptase on PAI and tPA expression in bEnd.3 cells. A. PAI and tPA mRNA level by RT-PCR.B. PAI and tPA mRNA level by real time RT-PCR. C. PAI and tPA protein levels by western blot. Tryptase increased tPA expression and inhibited PAI-1 expression at the mRNA and protein level in bEnd.3 cells. *P<0.01 vs. the mock and blank groups (n = 3).

Since endothelial cells play a pivotal role in the regulation of haemorrhage, we studied the effect of tryptase on the production of tissue plasminogen activator (tPA) and plasminogen activator inhibitor 1 (PAI-1) in vitro using bEnd.3 endothelial cell line. As shown in Fig. 7, tryptase increased tPA expression and inhibited PAI-1 expression at the mRNA and protein level in bEnd.3 cells, which was consistent with the in vivo effect of tryptase on atherosclerotic plaque.

## Discussion

The association of tryptase with angiogenesis and inflammation suggests that tryptase plays a causative role in intraplaque haemorrhage. The walls of the coronary artery are normally free of microvessels, except in the atherosclerotic plaques, where there are dense networks of capillaries that arise from the adventitial vasa vasorum and extend into the intimal layer of atherosclerotic lesions and other types of vascular injury [20]. Most of the intraplaque vasa vasorum are immature like tumour microvasculature and have abnormally high vascular endothelial permeability [21]–[23]. Microvascular disruption or leakiness may account for the accumulation of erythrocytes and may promote the conversion of a stable plaque to an unstable phenotype [1].

Numerous angiogenic factors have been identified in atherosclerotic plaques, which include vascular endothelial growth factor (VEGF), basic fibroblast growth factor (bFGF), tranforming growth factor-beta (TGF-beta), matrix metalloproteinases (MMPs), nitric oxide (NO), platelet-derived growth factor (PDGF), IL-8, platelet activating factor (PAF) and haem oxygenase-1[24]. Most of these factors have complex interactions with tryptase. Tryptase can stimulate endothelial cells to release interleukin IL-1, IL-6, IL-8, stem cell factor, TNFα and other inflammatory mediators [7], [25] and promote the chemotaxis of inflammatory cells, such as neutrophils and macrophages, into the plaque, which promotes the formation of new capillaries. Tryptase can activate matrix metalloproteinases. Some of these MMPs (e.g., MMP-9) further release angiogenic factors that are otherwise embedded in the extracellular matrix (ECM) [26], [27]. There are also studies showing that tryptase is involved in endothelial barrier dysfunction by activating endothelial PAR-2, resulting in protein kinase C (PKC) stimulation and elevation of Ca^2+^, which may cause increased vascular permeability [28], [29].

In our study, ApoE-/- mice were subjected to carotid collar placement surgery to create the atherosclerotic plaque haemorrhage model. Our results show that tryptase can contribute to atherosclerotic plaque angiogenesis and haemorrhage by regulating VEGF, PAI and tPA expression, while blood lipid levels were not affected by tryptase. Compound 48–80 also promoted plaque haemorrhage, which is consistent with Tang et al. 's report [15], but the effect is weaker than with tryptase overexpression.

PAI-1 is known to play a pivotal role in cardiovascular diseases, including arteriosclerosis and hypertension. Increased PAI-1 associated with reduced tPA was found in atherosclerotic lesions, especially during an acute cardiovascular event [30]. In contrast, Luttun et al. reported that PAI-1 knockout promoted advanced atherosclerotic plaque progression in apoE-/- mice [31], while tPA expression was elevated in advanced atherosclerotic plaques. Our results are consistent with the data from Luttun et al. We considered that the inconsistent reports of PAI and tPA expression in arteriosclerosis may be related to different periods of atherosclerotic plaque detection. At first, increased PAI in the early atherosclerotic plaque results in extracellular matrix accumulation and fibrin deposition and therefore promotes plaque formation [32]. During the development of atherosclerosis, an angiogenic response would thus be stimulated by hypoxia. At late stages of atherosclerosis, factors, such as tryptase, may contribute to the fragile microvascular bleeding by inhibiting PAI and increasing tPA. When acute cardiovascular events occur, increased microvascular bleeding may activate platelets by a positive feedback mechanism leading to large amount of platelet adhesion and aggregation, and PAI secretion. At this time, tPA is inactive, and the blood is in a "hypercoagulable" state [33].

The proliferation and migration of microvascular endothelial cells are important steps in the process of angiogenesis. Therefore, we studied the effects of tryptase on endothelial cell proliferation, migration and tube formation using mouse brain microvascular endothelial cells bEnd.3. The in vitro studies showed that tryptase increased bEnd.3 cell proliferation, migration and tube formation, which suggests that tryptase could promote angiogenesis. Tryptase inhibited PAI-1 expression, while increased tPA expression in bEnd.3 cells, which was consistent with the results of our in vivo studies. Currently, there is little data on the effect of tryptase on the expression of PAI-1 and tPA in endothelial cells. In tumour cells, it has been reported that the expression of PAI depends on PAR-2 activation [34]. Therefore, the mechanism of how tryptase regulates the expression of coagulation and fibrinolysis factors in endothelial cell needs further study.

In summary, tryptase plays an important role in atherosclerotic vascular inflammation by causing vascular endothelial dysfunction. Interestingly, tryptase can promote atherosclerotic plaque angiogenesis and haemorrhage. Our data suggest that tryptase may be a new target for the early treatment of vulnerable plaques.
